# The Bromide of Ethyl a Disappointing as Well as a Dangerous Anæsthetic

**Published:** 1880-08

**Authors:** Joseph C. Moore

**Affiliations:** Chicago


### ARTICLE VI.

*The Bromide of Ethyl a Disappointing as well as a Dangerous Anæsthetic.*

One of the chief reasons, perhaps, that medicine has not kept pace with other scientific advances, as is so often charged, is partially because experimentation in this field is liable to be attended with injury to life or health, and hence it is not every physician who feels heroic enough to put his theories to practical tests either upon himself or his patients.

While progressive persons in scientific medicine, as in other branches, are honored for their courage and praised for their independence, yet if they were not restrained by over-careful conservatism at the other extreme, they might do incalculable harm from too impetuous rashness. And again upon the other hand, ultra conservatism instead of simply standing still as it does, is kept by the progressive iconoclast from sliding positively backwards. Thus the two opposites, by reacting upon each other, produce the vast middle class whose maxim in effect seems to be expressed by the poet's familiar lines :

> "Be not the first by whom the new is tried,
> Nor yet the last to lay the old aside."

In medicine it is through the careful plodding examination of this middle class that accumulated clinical testimony is obtained, and each new remedy, discovery or invention is ultimately, impartially branded in accordance with its attested merits. It is to offer my own experience, and my opinion therefrom, upon the bromide of ethyl that I make this report, so that when the sum total of testimony is made up we shall know whether the bromide of ethyl should be placed upon the shelf of usefulness, or consigned to the limbo of therapeutic oblivion—that Hades for "vaunted specifics."

For some months past the journals have contained a number of articles upon the subject of this new anæsthetic. Much has been said in its praise, and some little in its condemnation. It was so recently brought forward that very little could be said against it, as few knew anything practically about it. But when so great an authority as Dr. Turnbull and so eminent a surgeon as Dr. Levis united their voices in sounding its praises, the profession as one man stood breathlessly still and silently drank in with a willing and a listening ear all that was to be learned regarding a substance which was claimed to be superior to chloroform or ether, and which filled a requirement long felt, but never before met. Dr. Levis concludes his recommendation in these unequvocal terms: "I express my conviction that it is practically the best anæsthetic known to the profession."

After such an unqualified indorsement the great mass of physicians congratulatingly said to themselves, "What an excellent thing is the bromide of ethyl!" while surgery smiled a sad quiet smile of blissful satisfaction.

The bromide of ethyl was said to act speedily; to produce little or no nausea; to pass off quickly; to influence respiration but little; to affect the circulation but slightly; and to cause but trifling general excitement, less even than chloroform; and last, though not least, to be free from danger. Free from danger!

Believing and expecting to realize at least a part if not the whole of these desirable conditions—a sort of anæsthesia made easy, as it were—a four-ounce bottle of John Wyeth & Bro.'s ethyl was procured, for which two dollars were paid—being a higher rate than is charged for the best chloroform—and following strictly the directions for administration as given by Dr. Levis, everything was in readiness for the trial, the result of which was to give such pleasure to the patient and such satisfaction to the operator.

The bromide of ethyl was given at separate times to two females, both being patients selected from the pratice of Dr. A. Reeves Jackson, and were deemed by Dr. Jackson and myself as good subjects upon which to fairly try the qualities of the anæsthetic.

It is not necessary that both cases should be recited, since one is a counterpart of the other, with the single exception that the first patient, after twenty minutes inhalation, exhibited bad symptoms and retained partial consciousness, so that the ethyl was abandoned for ether, which acted promptly and efficiently; while the second woman, the one I shall speak of on account of the sequel, knew what was going on around her after a trial of thirty-five minutes with ethyl, during which time alarming symptoms several times occurred. In this case also ether was substituted, which produced unconsciousness in about the usual time.

This second patient, whom I shall designate as Mrs. C., was to be operated upon by Dr. Jackson for uterine trouble. She was about 34 years of age, and not specially timid or nervous. She had eaten nothing upon the day set apart for the operation, and breathed the vapor of ethyl as well as persons commonly take anæsthetics; therefore the unfortunate result was no fault of hers. I gave the anæsthetic with unusual care and attention to every detail, therefore I think no just blame can be attached to me. Wyeth & Bro.'s goods are celebrated for their excellence, and this preparation of their house is specially commended by Dr. Levis himself, therefore it is evident that no blame should be attached to the chemist. It would seem, then, that the only fault was in the anæsthetic itself, and this I believe to be the fact.

But let us see how it acted, comparing the actual with the attributed results:

Its "speedy action" can hardly be said to be invariable, since twenty minutes in one case and thirty-five minutes in the other were consumed without producing unconsciousness.

So far as not producing much sickness is concerned, I never witnessed more intense and persistent nausea, or more racking retching by any anæsthetic before.

"Its effects pass of quickly." This I freely admit, "much too quickly." The very rapidity being a serous objection to its employment. A good prolonged fit of vomiting, such as the bromide of ethyl possesses the secret of occasioning—is very apt to be attended by a return to consciousness nearly before the patient is quite through the act.

"It only slightly affects the circulation." The pulse went up to a hundred and twenty, and did not fall much below, while the face became livid, the eyes deeply, dreadfully injected, and the veins of the head, face and neck were full and tense. So much congestion I never saw produced by an anæsthetic.

And as for the respiration, it was difficult and labored, and there was considerable rigidity, particularly about the chest walls, and at times approaching asphyxia seemed imminent.

If the general excitement caused by the bromide of ethyl is but "trifling," and what I witnessed was a trifling manifestation of its power proportionately reduced to suit female delicacy of structure and temperament, then I trust that I shall never be called upon to administer it to any full sized, vigorous, muscular mariner, in good fighting physical trim and in the usual blasphemous frame of mind.

And lastly it is said to be safe. Nothing that produces such conditions as were produced can ever be safe; nothing which presented such a picture of venous and pulmonary engorgement as was manifested in these women can possibly be safe. I have administered chloroform and ether in both hospital and private practice, and have been present on numerous occasions when these anæsthetics were given, but I never yet saw such dangerous and distressing symptoms produced as were developed under the bromide of ethyl. But further than this, let us inquire into its safety. It has been used but a short time—I think I may safely affirm that it has never been generally employed—and yet we have already two recorded deaths, which prove it to be, for the use it has received, one of the most dangerous anæsthetics known to modern medicine. One of the fatal cases, a woman, was reported by Dr. Hine, and the other, a young man of 18 years of age, occured in May last at the clinic of Dr. Levis—the guide, philosopher, and friend of "the best anæsthetic known to the profession." This last young man, the post-mortem disclosed, had "diseased brain, heart, lungs and kidneys." He had too much, for he had also a stone in the bladder, and—bromide of ethyl.

Now a word as to the comparative merits of the anæsthetics to show that there was no idiosyncrasy. It so happened that Mrs. C. required a secondary operation which was performed a few weeks after the one to which I have alluded. Ether was given, and in thirteen minutes from the commencement, without a single alarming symptom, without excitement, and without nausea, she was completely anæsthetized and ready for the operating table.

JOSEPH C. MOORE, M.D.

CHICAGO, July, 1880.

